# Longitudinal management and novel pharmacological interactions in a patient with HeFH, 21-OHD, and kawasaki disease: a 6-year clinical follow-up

**DOI:** 10.3389/fendo.2026.1823828

**Published:** 2026-04-23

**Authors:** Ying Zhang, Xin Yuan, Xiaohong Yang, Ruimin Chen

**Affiliations:** Department of Pediatric Endocrinology, Genetics and Metabolism, Fuzhou First General Hospital Affiliated with Fujian Medical University, Fuzhou Children’s Hospital of Fujian Medical University, Fuzhou, China

**Keywords:** 21-hydroxylase deficiency, familial hypercholesterolemia, hydrocortisone, kawasaki disease, pediatric, statins

## Abstract

**Background:**

Familial hypercholesterolemia (FH) and 21-hydroxylase deficiency (21-OHD) are both genetic disorders that significantly elevate cardiovascular risk in pediatric patients. Kawasaki disease (KD) further exacerbates this risk by causing coronary artery complications. This study reports a rare case of a child concurrently diagnosed with heterozygous FH (HeFH), 21-OHD, and KD-induced aortic aneurysm, highlighting a novel pharmacological interaction observed during treatment.

**Case presentation:**

A 15-day-old boy was initially diagnosed with 21-OHD following newborn screening. At age 1.1 years, he developed KD with coronary artery aneurysms. At 1.3 years, he presented with cutaneous xanthomas and severe hypercholesterolemia (LDL-C 13.0 mmol/L), leading to a diagnosis of HeFH (LDLR variant c.G2389A). Due to his exceptionally high cardiovascular risk, atorvastatin (5–10 mg/day) was initiated at age 2.6 years, followed by ezetimibe (5 mg/day) at age 3.7 years.

**Results:**

The combination therapy successfully reduced LDL-C levels to target goals without adverse effects on liver or muscle enzymes. As LDL-C levels decreased following statin therapy, the hydrocortisone dose required to manage 21-OHD increased from 7 mg/m²/day to 11.2 mg/m²/day.

**Conclusions:**

Early aggressive lipid-lowering therapy, including statins and ezetimibe, is safe and effective for high-risk FH infants under age 3. The observed negative correlation between LDL-C and hydrocortisone dosage suggests that statin-induced inhibition of cholesterol synthesis may interfere with adrenal steroidogenesis, requiring careful dose adjustment in patients with comorbid 21-OHD.

## Introduction

Familial hypercholesterolemia (FH, MIM#143890) is an autosomal-dominant genetic disorder primarily characterized by markedly elevated levels of low-density lipoprotein cholesterol (LDL-C). Heterozygous FH (HeFH) is a relatively common condition, affecting approximately 0.2% to 0.5% of the general population. Pathogenic variants in the low-density lipoprotein receptor (LDLR), apolipoprotein B (APOB), proprotein convertase subtilisin/kexin type 9 (PCSK9), or LDLR adaptor protein 1 (LDLRAP1) genes are the established genetic drivers of this condition (1). Due to the lifelong cumulative exposure to high LDL-C, patients with FH face a significantly increased risk of premature atherosclerotic cardiovascular disease (ASCVD). Evidence suggests that early diagnosis and the prompt initiation of LDL-lowering therapies are critical for improving long-term clinical outcomes (2).

Adding to the complexity of cardiovascular risk, 21-hydroxylase deficiency (21-OHD, MIM#201910) is a congenital adrenal hyperplasia (CAH) disorder characterized by impaired cortisol and aldosterone biosynthesis and subsequent adrenal androgen excess. Beyond the classic endocrine phenotype, the associated metabolic disturbances—including hypertension, obesity, and insulin resistance—further predispose affected individuals to accelerated vascular aging and early-onset cardiovascular complications (3, 4). Furthermore, Kawasaki disease (KD), a leading cause of acquired pediatric heart disease, involves systemic medium-sized vessel vasculitis that can result in life-threatening coronary or aortic aneurysms (5). The convergence of genetic dyslipidemia (FH), endocrine-mediated metabolic dysfunction (21-OHD), and acquired vascular injury (KD) creates an extreme risk profile that necessitates an aggressive and integrated therapeutic approach.

In this study, we report a six-year longitudinal follow-up of a pediatric patient concurrently diagnosed with HeFH, 21-OHD, and a KD-induced aortic aneurysm. Notably, the patient exhibited an exceptionally severe lipid phenotype and early-onset xanthomas, requiring the initiation of statin therapy at the remarkably young age of 2.6 years. Most importantly, our clinical observations revealed a novel inverse correlation between LDL-C levels and the required dosage of hydrocortisone replacement therapy—a pharmacological interaction that has not been previously documented. This case provides unique insights into the safety of early lipid-lowering interventions and the potential interplay between cholesterol homeostasis and adrenal steroidogenesis.

## Methods and results

In mid-2019, a 15-day-old male neonate presented following a positive newborn screening for 17-hydroxyprogesterone (17-OHP). Physical examination revealed a body weight of 3.22 kg, Tanner stage 1 breast development with areolar staining, a soft abdomen without cardiopulmonary abnormalities, Tanner stage 1 pubic hair, and scrotal hyperpigmentation. Biochemical analysis demonstrated potassium at 5.36 mmol/L, sodium at 127 mmol/L, total cholesterol (TC) at 5.94 mmol/L, LDL-C at 4.11 mmol/L, adrenocorticotropic hormone (ACTH) at 52 pg/mL, cortisol at 224 nmol/L, testosterone at 146 ng/dL, progesterone at 9.67 ng/mL, androstenedione at >10 ng/mL, and 17-OHP at 348 nmol/L. Following parental informed consent, Sanger sequencing identified a compound heterozygous variant in the *CYP21A2* gene (c.293-13C>G and c.T113A; p.I38N), with each parent carrying one variant. The patient was diagnosed with 21-OHD and hypercholesterolemia and was initiated on oral hydrocortisone at 1.1 mg every 8 hours (13 mg/m²/day) and fludrocortisone at 0.1 mg once daily. Regular monitoring showed that 17-OHP fluctuated between 0.1 and 11.3 nmol/L, testosterone between <2.5 and 22.66 ng/dL, and ACTH between <5 and 13 pg/mL, while progesterone, androstenedione, and electrolytes remained within normal ranges.

In July 2020, at 1.1 years of age, the boy was admitted to the Rheumatology Department with a three-day history of fever and a one-day history of rash and was diagnosed with Kawasaki disease (KD). Laboratory tests showed a TC of 9.88 mmol/L and LDL-C of 8.0 mmol/L, while cardiac ultrasound revealed dilated left and right coronary arteries (LCA internal diameter: 0.28 cm, Z-score +2.70 SDS; RCA internal diameter: 0.24 cm, Z-score +2.27 SDS) along with mild tricuspid and trace mitral regurgitation. He received high-dose intravenous immunoglobulin (4 g/kg total), aspirin, and dipyridamole, with methylprednisolone therapy for three days before transitioning to prednisone. By the seventh day of admission, his fever and clinical symptoms (conjunctival congestion and extremity swelling) had subsided, transaminases normalized, and ascites resolved. After two weeks, prednisone was transitioned to hydrocortisone at a dose of 1.1 mg three times daily (8 mg/m²/day). Repeat cardiac ultrasound showed a small aneurysm at the RCA ostium (0.41 cm, Z-score +6.94 SDS) and LCA dilatation (0.29 cm, Z-score +2.46 SD). Two months later, ultrasound showed persistent LCA dilatation (0.21 cm) and an RCA ostium diameter of 0.28 cm, with no other intracardiac abnormalities noted.

At 1.3 years of age, the patient was re-evaluated due to round, xanthochromic lesions on his wrists and ankles. Physical examination noted a height of 77.4 cm (-1.00 SDS) and weight of 8.8 kg (-1.41 SDS), while biochemistry revealed a TG of 0.91 mmol/L, TC of 14.56 mmol/L, and LDL-C of 13.0 mmol/L. Hormonal levels remained stable (17-OHP 2.3 nmol/L, testosterone <2.5 ng/dL, ACTH 23.5 pg/mL). Family history revealed a 30-year-old father with a TC of 13 mmol/L and a 50-year-old grandmother with a TC of 7.0 mmol/L, both asymptomatic without medication. Whole-exome sequencing confirmed the *CYP21A2* compound heterozygous variants and identified a heterozygous *LDLR* variant (c.G2389A: p.V797M), leading to a diagnosis of HeFH alongside 21-OHD and recovering KD.

Following dietary intervention, at 2.3 years of age, the boy was on hydrocortisone at 1.2 mg q8h (7 mg/m²/day) and fludrocortisone 0.1 mg qd. At 2.6 years of age, with a TC of 11.63 mmol/L and LDL-C of 9.64 mmol/L, and following Ethics Committee approval and parental consent, atorvastatin 5 mg qd was initiated. Ten days later, although liver and myocardial enzymes were normal and LDL-C had decreased to 8.33 mmol/L, his 17-OHP surged to 75 nmol/L. Consequently, the regimen was adjusted to atorvastatin 10 mg qd and hydrocortisone 2 mg q8h (11.2 mg/m²/day).

**Figure 1:**
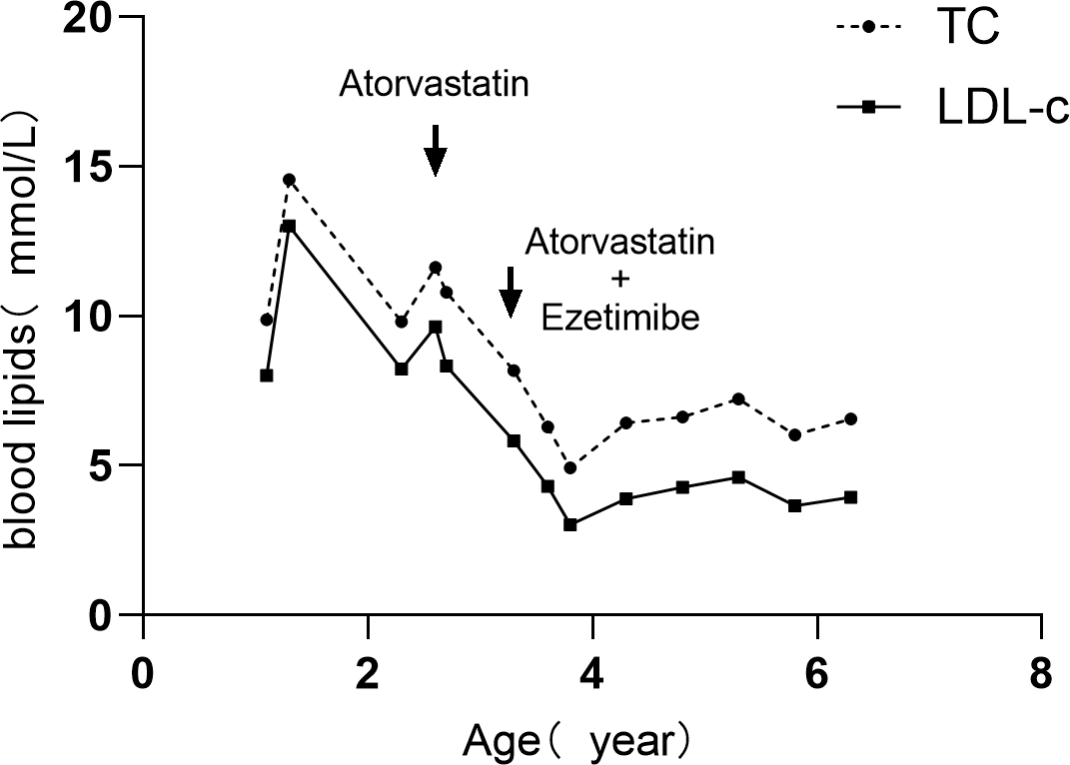
Serial serum TC and LDL-C Levels and lipid-lowering drug use during follow-up.

By October 2025, at the age of 6 years and 5 months, the patient’s height was 116.7 cm (-0.21SDS) and weight was 21.5 kg (-0.15SDS). He continued the combination regimen of atorvastatin 10 mg and ezetimibe 5 mg, while the hydrocortisone dose was adjusted to 3 mg every 8 hours (8.8 mg/m²/day). Laboratory results demonstrated an LDL-C of 3.94 mmol/L and a 17-OHP level of 9.41 nmol/L. His bone age was reassessed at 8 years and 1 month, indicating significant advancement. Cardiovascular ultrasonography revealed no significant coronary dilatation, with only mild aortic valve regurgitation. The data was shown in **Table 1** and **Figure 1**.

**Table 1:** Serial serum TC and LDL-C levels during follow-up.

Date	Age (years)	Treatment for FH	TC (mmol/L)	LDL-C (mmol/L)	Hydrocortisone (mg/m²/day)
2019-06-06	15 days	None	5.94	4.11	13.0
2020-07-14	1.1	None	9.88	8.00	8.0
2020-09-10	1.3	None	14.56	13.00	8.0
2021-09-06	2.3	None	9.80	8.22	7.0
2021-12-21	2.6	Atorvastatin 5 mg/day	11.63	9.64	7.0
2022-01-23	2.7	Atorvastatin 10 mg/day	10.79	8.33	11.2
2022-09-15	3.3	Atorvastatin 10 mg/day + Ezetimibe 2.5 mg/day	8.17	5.83	9.2
2023-01-10	3.6	Atorvastatin 10 mg/day + Ezetimibe 5 mg/day	6.29	4.30	8.5
2023-04-12	3.8		4.92	3.03	8.7
2023-10-29	4.3		6.42	3.89	8.2
2024-04-20	4.8		6.62	4.27	8.5
2024-10-19	5.3		7.23	4.61	8.5
2025-04-18	5.8		6.03	3.65	8.6
2025-10-25	6.3		6.55	3.94	8.8

FH, familial hypercholesterolemia; TC, total cholesterol; LDL-C, low-density lipoproteins cholesterol.

## Discussion

It is well-established that cardiovascular risk factors present during childhood have a profound and well-documented association with the occurrence of adverse cardiovascular events in adulthood. A variety of risk factors that accelerate atherosclerotic progression can be identified in pediatric populations, including dyslipidemia, obesity, hypertension, and diabetes mellitus (6). Among these, FH represents a common and significant genetic cause of early-onset coronary heart disease. In patients with FH, continuous exposure to elevated levels of LDL-C leads to persistent cholesterol deposition within the arterial walls and triggers vascular inflammation, which eventually progresses to advanced atherosclerosis, particularly within the coronary arteries and the aorta (7).

This study describes the comprehensive diagnosis and longitudinal six-year follow-up of a young boy presenting with a rare and complex combination of HeFH, 21-OHD, and an aortic aneurysm resulting from KD. While both HeFH and 21-OHD have clear genetic etiologies rooted in specific gene mutations, the exact pathogenesis of KD—an acute immune-mediated vasculitis—remains largely elusive. Given the clinical overlap in this patient, we investigated whether 21-OHD or FH might serve as a predisposing risk factor for the development of KD. However, such occurrences have only been documented in a limited number of case reports (8, 9). While 21-OHD has been linked to an increased risk of certain autoimmune disorders, such as Grave’s disease, no statistically significant increase in the incidence of KD has been observed in the 21-OHD population (10). Furthermore, research by Chen X et al (11) into the lipid profiles of patients with KD found that the disease is typically characterized by elevated triglyceride levels rather than TC, suggesting that high cholesterol levels do not have a significant pathogenic association with the onset of KD.

A remarkable clinical feature in this boy was that his cholesterol levels were significantly higher than those seen in average HeFH patients, and he developed visible xanthomas at an exceptionally young age. This led to the question of whether 21-OHD might be a contributing factor to his extreme hypercholesterolemia. Numerous studies have demonstrated that CAH is associated with an increased risk of metabolic complications, including weight gain, impaired insulin sensitivity, hypertension, endothelial dysfunction, early atherosclerotic vascular changes, and left ventricular diastolic dysfunction (12–14). Despite these general risks, specific lipid analyses by Subbarayan A et al (15) in children and adolescents with 21-OHD revealed that only 9.5% had high plasma triglycerides and a mere 3% had elevated plasma cholesterol. Other studies involving adult males with 21-OHD confirmed no significant difference in cholesterol levels compared to healthy controls (16). Moreover, animal models have indicated that adrenalectomy is actually associated with a decrease in plasma lipid levels; in LDL receptor knockout mice, cholesterol levels fell rather than rose post-adrenalectomy. Specifically, total cholesterol and triglycerides were 22% and 29% lower in adrenalectomized mice compared to controls (17).

These findings strongly suggest that 21-OHD itself does not cause a significant increase in cholesterol levels. Instead, the severe phenotype is better explained by the specific nature of the identified genetic variant. According to the functional characterization by Shu et al. (18), this c.2389G > A substitution is not a typical missense mutation; rather, it leads to a splicing error resulting in the deletion of exon 16 in the LDLR mRNA. This structural alteration caused the mutant receptor protein to be primarily retained within the Golgi apparatus, severely impeding its transport and expression on the cellular membrane. This profound deficit in LDL clearance effectively mimics the clinical severity typically reserved for homozygous FH. Consequently, this high degree of functional impairment necessitated early and aggressive lipid-lowering intervention, despite the patient’s heterozygous status.

An intriguing observation during this child’s long-term management was that his required hydrocortisone dose remained lower than that of most pediatric patients over a prolonged period. Before the initiation of statin treatment, his maintenance dose was approximately 7 mg/m²/day. However, following the introduction of statin therapy, the necessary dose had to be increased to 11.2 mg/m²/day to maintain hormonal control. Based on these observations, we hypothesize that there is a negative correlation between the patient’s cholesterol levels and his required hydrocortisone dosage. A plausible mechanism for this phenomenon is that statins interfere with HMG-CoA reductase-mediated endogenous cholesterol synthesis, which in turn limits the availability of cholesterol as a substrate for adrenal steroidogenesis, thereby altering glucocorticoid secretion and increasing the external requirement for hydrocortisone (19). Furthermore, recent evidence highlights the role of oxysterols in modulating adrenal function. Statins have been shown to reduce levels of 27-hydroxycholesterol (27-HC), a potent endogenous ligand for the liver X receptor (LXR), particularly in pediatric FH populations (20). Diminished 27-HC levels may lead to reduced LXR signaling, which is critical for regulating intracellular cholesterol mobilization and transport within the adrenal cortex. This secondary mechanism—impaired substrate mobilization—may act synergistically with reduced total cholesterol availability to further suppress residual adrenal capacity in CAH patients. The clinical implication of this “cholesterol-adrenal axis” interaction is significant. It suggests that in patients with comorbid adrenal insufficiency and dyslipidemia, aggressive lipid-lowering interventions may alter steroid metabolism or requirements. Clinicians should therefore perform rigorous hormonal monitoring when initiating or intensifying statin therapy in this high-risk population to ensure timely dose adjustments and prevent potential adrenal crises.

The management of HeFH remains a significant clinical challenge, as many patients are diagnosed late, are undertreated, and frequently fail to achieve guideline-recommended LDL-C targets (21, 22). Current therapeutic strategies for HeFH involve a combination of rigorous dietary interventions and pharmacotherapies, including statins, ezetimibe, and newer agents such as PCSK9 inhibitors or evinacumab (23). Nutrition and dietary interventions can reduce LDL-C by up to 3-22% (24), but if target levels are not reached after at least three months of lifestyle modification, pharmacological intervention becomes necessary. Ezetimibe is widely utilized as a well-tolerated second-line therapy, either alone or in combination with statins (25).

While international recommendations generally suggest initiating statin therapy in HeFH patients between the ages of 6 and 8, the clinical circumstances of this patient were exceptional. Given his dangerously high cholesterol levels, the early appearance of xanthomas, and his history of a KD-induced aortic aneurysm, atorvastatin therapy was initiated at the age of 2.6 years following ethical approval and comprehensive parental counseling. Statins disrupt HMG-CoA reductase and effectively lower plasma LDL-C levels, but their long-term effects in very young children are not as extensively documented as in adults. A systematic review (26) evaluating statin efficacy in children with FH found that these medications significantly reduce LDL-C levels and are generally safe and well-tolerated with minimal adverse effects. In these populations, statins achieved LDL-C reductions of 25% to 43.8%, with potential side effects—such as elevated liver enzymes or muscle enzymes—being rare. Throughout the extended treatment period for this child, rigorous clinical and laboratory monitoring revealed no evidence of hepatotoxicity, myopathy, or other treatment-related adverse effects.

Despite the initial success of statin monotherapy, his LDL-C levels remained above the target established by current guidelines, which recommend either a 50% reduction from baseline or an absolute level below 3.5 mmol/L (27). Consequently, after nine months of statin therapy, ezetimibe was added to the regimen, which successfully brought his cholesterol levels within the target range without any adverse events. Regular cardiovascular monitoring via carotid and cardiac color Doppler ultrasound confirmed that no new atherosclerotic changes developed during this period. As his cholesterol levels were successfully lowered, we again noted the concomitant need for an increased hydrocortisone dose.

## Limitations of this study should be acknowledged

First, as this is a single case report, the observed negative correlation between LDL-C levels and hydrocortisone dosage requires further validation through larger cohort studies or multicenter registries. Second, although we hypothesize that statin-induced cholesterol reduction interferes with adrenal steroidogenesis, we did not perform dynamic adrenal function tests (such as ACTH stimulation tests) at each dosage adjustment to definitively map the metabolic interplay. Lastly, while the 6-year follow-up provides valuable longitudinal data, the long-term impact of early-onset statin therapy (initiated at age 2.6) on the patient’s final adult height and pubertal development remains to be seen, especially given the current advancement in bone age.

## Conclusion

We present a complex case of a pediatric patient diagnosed with 21-OHD, HeFH, and KD complicated by a coronary artery aneurysm, managed over a comprehensive six-year follow-up period. Notably, the patient exhibited LDL-C levels significantly higher than those typically observed in pediatric HeFH populations. A primary clinical finding of this study is that the initiation and intensification of lipid-lowering therapy led to a concomitant increase in the required hydrocortisone replacement dosage to maintain hormonal stability. This phenomenon—where a reduction in circulating cholesterol levels appears to necessitate higher exogenous glucocorticoid support—has not been previously documented in medical literature. This clinical interplay suggests that in patients with co-existing adrenal insufficiency, aggressive lipid-lowering interventions may alter steroidogenesis or steroid metabolism, thereby requiring compensatory upward adjustments of glucocorticoid doses. Further mechanistic studies are warranted to elucidate the metabolic pathways underlying this observation and to optimize integrated management strategies for this high-risk population.

## Data Availability

The original contributions presented in the study are included in the article/supplementary material. Further inquiries can be directed to the corresponding author.
